# Functional and Mechanistic Insights into Plant VQ Proteins in Abiotic and Biotic Stress Responses

**DOI:** 10.3390/plants14243855

**Published:** 2025-12-17

**Authors:** Lili Zhang, Yi Wang, Zhiyong Ni, Yuehua Yu

**Affiliations:** 1 College of Agronomy, Xinjiang Agricultural University, Urumqi 830052, China; zlili9965@126.com; 2 College of Life Sciences, Xinjiang Agricultural University, Urumqi 830052, China; wangy@xjau.edu.cn

**Keywords:** VQ proteins, stress adaptation, WRKY transcription factors, molecular mechanisms, CRISPR-based editing

## Abstract

Valine-glutamine motif proteins (VQ), plant-specific transcriptional co-regulators harboring the conserved FxxhVQxhTG motif, play pivotal roles in coordinating plant stress adaptation through dynamic interactions with WRKY transcription factors (WRKY), mitogen-activated protein kinases (MAPKs) cascades, and hormone signaling pathways. Evolutionary analyses reveal the characteristics of their evolutionary protection and ancient origin, with lineage-specific expansion via genome duplication events. Structurally, compact genes lacking introns and the presence of intrinsic disordered regions (IDRs) facilitate rapid stress responses and versatile protein interactions. Functionally, VQ proteins orchestrate abiotic stress tolerance (e.g., drought, salinity, temperature extremes) by modulating reactive oxygen species (ROS) homeostasis, osmotic balance, and abscisic acid/salicylic acid (ABA/SA)-mediated signaling. Concurrently, they enhance biotic stress resistance via pathogen-responsive WRKY-VQ modules that regulate defense gene expression and hormone crosstalk. Despite advances, challenges persist in deciphering post-translational modifications, tissue-specific functions, and cross-stress integration mechanisms. Harnessing CRISPR-based editing and multi-omics approaches will accelerate the exploitation of VQ genes for developing climate-resilient crops. This review synthesizes the molecular architecture, evolutionary dynamics, and multifunctional regulatory networks of VQ proteins, providing a roadmap for their utilization in sustainable agriculture.

## 1. Introduction

VQ proteins are a class of plant-specific transcriptional co-regulators characterized by a conserved VQ-motif, typically within the canonical FxxhVQxhTG sequence pattern (where x denotes any amino acid and h a hydrophobic residue), with the VQ core motif exhibiting extreme conservation [1,2]. While the VQ domain is highly conserved across species, flanking regions exhibit considerable variability, contributing to functional diver-sity functional diversity [3]. Most VQ genes are intronless, encode relatively short proteins (<300 amino acids), and are predominantly nuclear-localized [2], features consistent with a role in rapid transcriptional regulation [4]. Genomic studies reveal that VQ gene families are unevenly distributed across plant chromosomes and can be phylogenetically classified into multiple subgroups (typically 7–9), with members within subgroups sharing similar gene structures and motif patterns [5]. This organized complexity underscores the role of VQ proteins as integral components of plant regulatory networks [2,6].

The primary research significance of VQ proteins lies in three areas. First, they function as key co-factors, often interacting with WRKY transcription factors to modulate stress signaling. Examples include potato *VQ31* influencing pathogen defense [7], rice OsVQ25 balancing immunity and growth via interactions with OsWRKY53 and an E3 ligase [8], tomato *SlVQ10* enhances SlWRKY51’s activation of D-1-pyrroline-5-carboxylate synthetase (P5CS) by promoting proline accumulation to improve cold tolerance [9], and sweet potato VQ4 interacts with IbWRKY2 protein to regulate genes related to ABA signaling, proline biosynthesis, and ROS scavenging, thereby enhancing drought and salt stress tolerance [10]. Second, the differential expression of VQ genes under various stressors (e.g., polyethylene glycol, salt, and ABA) highlights their potential as targets for stress-resistance breeding [2]. Additionally, VQ 1/10 can form homo- or heterodimers and interact with other regulators in Arabidopsis to coordinately modulate plant growth and stress responses. This synergy underscores the diversity and complexity of VQ gene functions, indicating that precise coordination among multiple genes and regulatory pathways is essential for plants to cope with varying environmental stresses [11]. Third, their compact gene structure makes them amenable to genetic manipulation, as demonstrated by CRISPR-mediated studies in tomato and rice that altered disease resistance phenotypes [12]. Modulation of *SlVQ15* reduces tomato resistance to *Botrytis cinerea*, and knockout of *OsVQ8* in an *OsWRKY10* overexpression rice line results in higher disease resistance, indicating an antagonistic relationship between the two and negatively regulating resistance to bacterial wilt and rice blast [13].

Although VQ proteins are implicated in ROS homeostasis and hormone pathways [14,15,16], their interaction mechanisms with partners like MAPKs remain incompletely understood [17]. Future research integrating multi-omics data is needed to fully exploit VQ proteins in designing stress-resilient crops [18,19].

## 2. Molecular Characteristics and Classification of VQ Protein

### 2.1. Molecular Structure Characteristics of VQ Protein

Nearly 75% of VQ genes lack introns, a compact genomic architecture suggestive of a functional adaptation for rapid response to environmental cues [20]. Promoter analyses further reveal an abundance of cis-acting elements associated with stress responses and growth/development, indicating potential for diverse regulatory roles [17]. Phylogenetically, the VQ gene family has expanded via different mechanisms in angiosperms (e.g., segmental duplication) and gymnosperms (e.g., tandem duplication), with purifying selection playing a dominant role in their evolution [1,21]. This strong selective pressure explains the high conservation of the core VQ motif across long evolutionary timescales. VQ proteins are plant-specific regulators [2,20,22], typically small (<300 amino acids) [1,3,20]. Their tertiary structure often includes IDRs, conferring flexibility for interactions with multiple partner proteins [23]. Notably, variable amino acid motifs upstream of the conserved VQ motif may determine interaction specificity [24]. Multi-species comparisons show that the VQ domain itself is extremely conserved within plants, while flanking sequences are more variable [1]. This conservation underscores its fundamental role, either independently or via transcription factor interactions, in regulating growth, development, and responses to biotic/abiotic stresses [24,25].

Within protein interaction networks, VQ proteins function as key hubs. They not only bind WRKY transcription factors (particularly Groups I and IIc) via their conserved motif [25,26] but also interact with signaling components like MAPKs [27], forming complex regulatory networks. Structural biology studies suggest the VQ-WRKY domain may rely more on structural features than specific sequences [23,28]. This multivalent interaction capability allows VQ proteins to integrate multiple signaling pathways, coordinating plant growth, development, and stress adaptation [20]. In short, their role as pivotal molecular adapters stems from distinctive structural features: (1) a compact gene structure, (2) a highly conserved yet context-variable core VQ motif, (3) predominant nuclear localization; (4) interaction flexibility provided by IDRs; and (5) functional differentiation reflected in subgroup classification.

### 2.2. Classification of VQ Protein

Phylogenetic analyses typically classify VQ proteins into 7–10 subgroups [29,30]. Members within a subgroup share similar motif composition [21] and exhibit conserved subcellular localization patterns, with most being nuclear [1,20], consistent with their functional role as transcriptional co-regulators. Although the VQ motif is highly conserved, at least 10 variant forms have been identified across species [31,32]. Subgroup composition varies among plants; for instance, 23 VQ genes in quinoa fall into 3 subgroups [33], 31 in *coix* into 7 subgroups, and 21 in rubber tree into 6 subgroups [34]. This classification, based on phylogenetic topology, is often supported by conserved gene structures (exon-intron patterns) and protein motif distributions within subgroups [6,35]. For instance, members within the 10 cotton VQ clades share similar 3D structures and motifs [30], while tea plant CsVQ proteins from 5 groups, with an evolutionary trajectory aligning with plant evolution [36]. Subgroup classification has functional implications: phylogenetically related subgroups often participate in similar processes. In tobacco, members of subgroups II, IV, V, VI, and VIII show significant responses to various hormones [25]. Interaction specificity is also subgroup-associated; soybean VQ proteins bind selectively to Group I and IIc WRKYs, a pattern likely shaped by co-evolution [26].

Cross-species comparative phylogenetics indicate an ancient origin for VQ genes, with family members present in fungi, lower animals, and bacteria, suggesting an evolutionary history predating land plants [37]. The VQ motif is highly conserved across kingdoms, with strict sequence consistency largely confined to the core domain. This conservation likely reflects true homology. Family expansion primarily occurs via tandem and dispersed duplications, with the motif-encoding regions under purifying selection, indicating evolutionary preservation from a common ancestor [37]. In angiosperms, whole-genome duplication (WGD) events have significantly driven VQ family expansion, as seen in rice and *Salix suchowensis* (Bamboo basket willow) [5,16]. Wheat VQ gene expansion is also linked to recent bursts of tandem and dispersed duplications [16]. Syntenic analyses confirm clear orthologous relationships for VQ genes from rice to wheat, a conservation potentially linked to their core role in stress response [16]. Thus, phylogenetic and evolutionary analyses provide the molecular basis for subgroup classification and clues to functional differentiation during plant adaptation.

## 3. Role of VQ Protein in Abiotic Stress

VQ proteins are crucial modulators of abiotic stress tolerance, enhancing or reducing plant resilience primarily by fine-tuning hormone signaling (e.g., ABA, SA) and antioxidant systems. A representative example is wheat *TaVQ4-D*, whose overexpression significantly improved drought tolerance in transgenic *Arabidopsis* and wheat. Transgenic plants exhibited enhanced antioxidant responses (elevated SOD activity and proline content, decreased malondialdehyde, upregulated ROS-scavenging genes) and stress-related gene expression, whereas silenced plants showed the opposite phenotype [17]. Conversely, overexpression of apple *MdVQ37* altered leaf development and SA balance under drought but ultimately reduced drought tolerance due to diminished photosynthetic capacity and enzyme activity, suggesting VQ proteins can function antagonistically or synergistically in drought response [38]. Similarly, quinoa *CqVQ13* was strongly upregulated under drought, and its nuclear localization suggests a role in coordinating stress-responsive gene networks via transcription factor regulation [33].

Under salt stress, VQ proteins enhance tolerance by regulating ion homeostasis and osmoprotect synthesis. In *Arabidopsis* overexpressing moso bamboo *PeVQ28*, salt stress induced nine ABA biosynthesis-related genes, while suppressing salt-sensitive genes, leading to reduced Na^+^ accumulation and maintained cell membrane stability [39]. Poplar VQ1 protein boosts salt tolerance by activating ABA/SA signaling pathways, promoting proline synthesis gene expression, and modulating catalase activity to maintain ROS homeostasis [15]. Similarly, potato *StVQ31* enhances salt tolerance in transgenic *Arabidopsis* by upregulating antioxidant enzyme activity (Catalase, CAT; SOD; Peroxidase, POD), reducing ROS accumulation (O^2−^ and H_2_O_2_), and activating salt-responsive genes [7]. Importantly, VQ protein interaction with transcription factors can form a multi-layered network. In tomato, SlWRKY57 negatively regulates salt tolerance. Its transcriptional repression activity is finely tuned through competitive binding with SlVQ16 and SlVQ21. This module also interfaces with Jasmonate (JA) signaling by binding Jasmonate ZIM-domain (JAZ) repressors, dynamically influencing salt tolerance [40].

Response to temperature stress involves distinct mechanisms. For heat stress, studies overexpressing apple *MdVQ37* conferred a heat-sensitive phenotype, linked to reduced enzyme activity, photosynthetic capacity, and endogenous SA levels, disrupting SA-dependent signaling [22,41]. Moreover, the GO and KEGG pathway analyses revealed that transcription factor activity and plant hormone signaling pathways were differentially affected and enriched in the transgenic lines. Overexpression of *MdVQ37* reduced endogenous SA levels by regulating the expression of SA catabolism-related genes, ultimately disrupting the SA-dependent signaling pathway under hormone stress. Mechanistically, the decline in SA may have diminished the efficiency of the antioxidant defense system, repair of damage metabolism, regulation of plant hormone signaling pathways, or the repair of oxidative damage caused by high temperatures. Similarly, heterologous expression of tomato *SlVQ6* reduced thermotolerance in *Arabidopsis* and downregulated stress-responsive genes [22]. For cold stress, tomato SlVQ10 interacts with transcription factor SlWRKY51 to enhance its activation of the proline synthesis gene *P5CS*, promoting proline accumulation and cold tolerance [9]. However, the precise regulatory networks of VQ proteins in temperature stress, especially their spatiotemporal specificity and crosstalk with other pathways, require further investigation [4,42].

**Figure 1:**
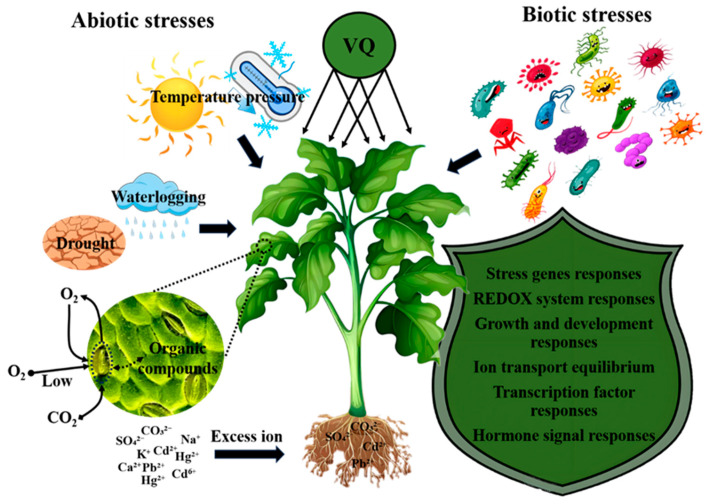
Schematic model illustrating the versatile regulatory roles of VQ proteins in plant responses to diverse abiotic and biotic stresses.‌ Under abiotic stresses (drought, salinity, osmotic imbalance, temperature extremes, hypoxia/waterlogging, heavy metals), VQ proteins function as pivotal signaling hubs. They interact with transcription factors via their conserved motif and modulate signaling pathways to orchestrate complex defense responses. This involves fine-tuning hormone signaling, enhancing antioxidant systems, regulating ion homeostasis, promoting osmoprotectant synthesis, and modulating stress-responsive gene networks. VQ proteins also integrate REDOX/NO signaling and maintain protein homeostasis under specific stresses like hypoxia and heavy metals. Conversely, in biotic stress triggered by pathogens, VQ proteins act as molecular switches within the PAMP-triggered immunity pathway. They modulate defense by forming VQ-WRKY complexes, influencing MAPK signaling, regulating ROS metabolism, and coordinating hormone cross-talk, ultimately impacting the expression of pathogenesis-related (PR) genes and disease resistance.

VQ proteins also regulate hypoxia responses. *Arabidopsis AtVQ10* acts as a potential node integrating redox signals; its expression is induced by hypoxia, NO, and oxidative stress. Hypermorphic vq10-H mutants and overexpressors showed reduced submergence but enhanced oxidative stress tolerance and decreased NO sensitivity, indicating VQ10 modulates hypoxia response via redox balance [43]. This likely involves WRKY interaction, as genes within the VQ-WRKY network (including oxygen-sensing transcription factors) show altered expression under hypoxia [44]. In short, under abiotic stress, VQ proteins enhance plant adaptability by integrating hormone signaling (ABA, SA, JA), ROS/NO homeostasis, and transcriptional networks (Figure 1). Their precise downstream targets and pathways, however, need further functional validation.

## 4. Function of VQ Protein in Biological Stress

**Figure 2:**
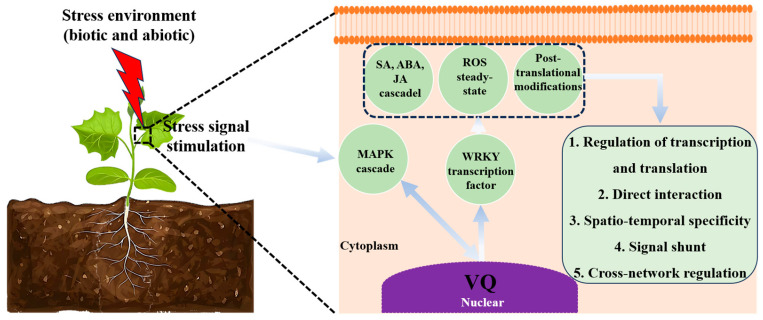
Integrated molecular network of VQ protein functionality in plant stress adaptation. ‌VQ proteins orchestrate abiotic and biotic (pathogen) stress responses through dynamic interactions with WRKY, MAPK cascades, hormone signaling, and ROS homeostasis. Their roles also extend to epigenetic regulation, enabling transcriptional memory and inducible plasticity.

Nuclear localization of VQ proteins provides a spatial advantage for forming interaction networks with transcription factors, facilitating their role in defense against pathogens [20]. The VQ-WRKY complex represents a central regulatory node: A representative example is wheat TaVQ22 binds the DNA-binding domain (DBD) of TaWRKY19-2B, suppressing its transcriptional activity, modulating ROS homeostasis, and negatively regulating the defense against the sharp eyespot pathogen [14]. Rice OsVQ14 and OsVQ32 participate in immune regulation against *Xanthomonas oryzae* pv. *Oryzae* (*Xoo*) via the MAPK pathway [27]. Conversely, apple *MdVQ17* overexpression increased susceptibility to *Glomerella* leaf spot (GLS). By interacting with MdWRKY17, it modulated SA accumulation and pectinase activity, promoting pathogen susceptibility [45]. VQ proteins can exhibit functional pleiotropy; poplar *VQ1* overexpression enhanced both salt tolerance and pathogen resistance in *Arabidopsis* via ABA and SA pathway activation [15]. Despite functional divergence across species, a core mechanism is conserved: variable upstream motifs determine WRKY-binding specificity, leading to regulation of stress-responsive genes [24]. Integrating current evidence, a functional framework emerges: Pathogen-associated molecular patterns (PAMPs) perception triggers MAPK cascade → VQ proteins interact with WRKYs/MAPKs → modulate ROS metabolism/hormone signaling (SA/ABA/JA) → activate defense effector molecules like PR genes [14,27,46]. Although VQ families are identified in crops like cotton [30] and tea [36], studies on their expression patterns and functions under various stimuli remain limited [4,47]. Recent focus on their intrinsically disordered protein (IDP) nature [23] highlights structural plasticity as a potential target for engineering broad-spectrum disease resistance. In summary, VQ proteins act as molecular switches in biotic stress via a “signal perception–partner recruitment–transcriptional regulation” cascade [20] (Figure 2). Future work should elucidate the structural basis of VQ interactions and integrative mechanisms under combined stresses (e.g., drought-pathogen) [14,20].

## 5. Action Mechanism and Regulatory Networks

Plant VQ proteins exert pivotal functions in growth, developmental, and stress adaptation primarily by orchestrating interaction networks with WRKY and MAPK cascades. They modulate WRKY transcriptional activity by binding their DBDs. In *Arabidopsis*, sigma factor binding protein 1 (SIB1) forms a stable complex with the DBD of WRKY33 minimal interaction sequence (the VQ motif and its preceding sequence), though the structural basis needs elucidation [23]. Wheat TaVQ22 inhibits TaWRKY19-2B’s transcriptional activation via its VQ motif, regulating ROS homeostasis and defense against *Fusarium pseudograminearum* [14]. The VQ-WRKY module also participates in hormone signaling; rice *OsVQ25* suppresses *OsWRKY53* activity, regulating plant immunity and BR signaling [8]; the apple MdVQ17-MdWRKY17 interaction mediates infection response via SA pathway regulation [45], and the MdVQ37-MdWRKY100 module defends against GLS by modulating SA accumulation [46] and promotes salt tolerance by regulating Na^+^/K^+^ homeostasis and ROS clearance [48]. This network exhibits dual regulatory characteristics. In tomato, SlVQ16 and SlVQ21 competitively bind SlWRKY57, antagonistically regulating its expression activity and differentially influencing salt tolerance. This module further engages JA signaling via JAZ proteins [40]. In *Arabidopsis*, WRKY33 and WRKY57 competitively bind SIB1/SIB2 VQ motifs, regulating JAZ1/5 expression to fine-tune JA signaling and WRKY33-mediated resistance to *Botrytis cinerea* [49].

VQ proteins also interact with MAPKs as phosphorylation substrates, affecting their stability and function [50]. Rice OsVQ1 interacts with and inhibits OsMPK6. Pathogen infection downregulates *OsVQ1* via the OsMPKK10.2-OsMPK6-OsWRKY45 cascade, forming a negative feedback regulatory loop involved in disease resistance and flowering [51]. Upon *Xoo* infection, OsVQ14 and OsVQ32 interact with and are phosphorylated by OsMPK4. Overexpression of the upstream kinase OsMPKK6 enhances *Xoo* resistance and increases OsVQ14/32 phosphorylation, indicating they act as signaling components in the OsMPKK6-OsMPK4 cascade [27]. Under abiotic stress, tomato SlVQ6 (which reduces thermotolerance) interacts with and is phosphorylated by SIMPK1, playing roles in drought, heat, and salt responses [22]. Similarly, wheat TaVQ4-D phosphorylation by MAPK3/6 enhances drought resistance by boosting antioxidant enzymes and stress gene expression [17]. Furthermore, the MAPK cascade pathway in rice can also mediate changes in VQ proteins (e.g., phosphorylation of rice OsVQ8), which in turn influence WRKY10 to participate in the co-expression and activation of specific genes involved in the biosynthesis of diterpenoid phytoalexins (DPs), thereby enhancing the accumulation of DPs and improving resistance to blast disease and bacterial leaf blight [13].

These interactions form a three-tiered network: upstream signals activate VQ proteins via MAPKs; midstream VQ-WRKY integrate stress signals; downstream effects regulate the antioxidant system, hormone synthesis, and defense genes. Cross-regulation is evident, as the same VQ protein (e.g., TaVQ4-D) can respond to both drought and pathogen stresses, while different VQ members (e.g., OsVQ8 and OsVQ25) may have antagonistic roles within the same pathway [1]. The network extends to other regulatory layers. In immunity, rice OsVQ25 fine-tunes the immunity-growth balance by promoting OsWRKY53 degradation via an E3 ubiquitin ligase pathway [8]. Thus, VQ proteins function as multifunctional “adaptors”: they alter transcription factor DNA-binding capacity (VQ-WRKY) [23,40], undergo post-translational regulation (e.g., ubiquitination) [8], and influence kinase activity (e.g., MAPK inhibition) [51].

## 6. Research Challenges and Prospectives

Research on plant VQ protein stress resistance mechanisms encounters several challenges. First, the specific molecular mechanisms and regulatory networks of VQ interactions with WRKYs and MAPKs remain unclear. For instance, the basis for selective binding of soybean VQ proteins only to Group I/IIc WRKYs needs investigation [26]. Second, studies on post-translational modifications (PTMs: phosphorylation, ubiquitination, acetylation) of VQ proteins and their functional impacts are severely lacking [52]. Future work should employ integrated proteomics strategies (e.g., modification-specific enrichment coupled with label-free quantitative mass spectrometry) to map PTM networks and cross-regulatory mechanisms (e.g., phosphorylation regulating ubiquitination) [8]. Rice OsVQ14 and OsVQ32, involved in MAPK signaling and disease resistance, are prime candidates for such PTM studies [27]. Third, the functional conservation and specificity of VQ proteins across whole plant species are unclear. While VQ families are identified in many species [30,36,53], functional validation is insufficient. For example, potato VQ functions are poorly studied [7], and tomato SlVQ6 overexpression reduced thermotolerance in *Arabidopsis* [22]; the molecular basis for such differences is unknown. Fourth, how VQ proteins integrate multiple stress signals is unresolved. Although VQ genes respond to diverse stresses [4,41], the coordination mechanisms are unclear. Finally, research on tissue-specific expression and developmental stage-specific regulation of VQ proteins is also relatively lacking [54], hindering a full-life-cycle understanding of their functions.

Future research should focus on: (1) Systematic analysis of VQ protein 3D structures and functional domains, especially the conserved FxxhVQxhTG motif mechanism [20,39]; (2) Employing comparative genomics and evolutionary analysis to reveal family origin and expansion patterns [37]; (3) In-depth studies on VQ PTM networks and their effects on protein stability and function [31,52]; (4) Utilizing VQ genes as targets for molecular breeding [31]. Overexpression of TaVQ4-D enhanced drought tolerance [17], and TaVQ14 improved seed germination under salt/drought stress [55]; such genes can serve as molecular markers. Gene editing (e.g., CRISPR-Cas9) can create VQ gene gain/loss-of-function germplasm [33,56]; (5) Constructing VQ regulatory network models integrating hormone signaling [15,39] to develop multi-stress resistance crops; (6) Methodologically combining multi-omics technologies, high-resolution microscopic imaging, and protein interaction network analysis to systematically reveal VQ’s core role [1,18]; (7) Strengthening functional studies in horticultural crops and trees (e.g., apple, rubber tree) [34,45,57] for practical applications.

## 7. Conclusions

Plant VQ proteins are plant-specific transcriptional co-regulators that are central to mediating responses to adverse environments. Through the conserved FxxhVQxhTG motif, they interact with diverse transcription factors and signaling components, forming sophisticated regulatory networks that orchestrate stress responses [20]. They exhibit dual regulatory functions, activating hormone pathway genes (e.g., ABA, SA) [15,22] and modulating organ development via phenotypic plasticity for environmental adaptation [38]. Phylogenetically, the VQ gene family is highly conserved across land plants [37]. The enrichment of stress-responsive *cis*-elements in their promoter, coupled with species-divergent expression profiles [4,31], makes them a valuable reservoir for molecular breeding. CRISPR/Cas9-based genome editing and targeted engineering of VQ modules offer innovative strategies to overcome conventional breeding limitations and develop germplasm with enhanced stress resilience and agronomic traits. However, it is important to emphasize that when evaluating the trade-off between yield increase and pathogen resistance, traditional disease-resistant breeding often comes with growth suppression, leading to yield loss, creating an inherent trade-off effect. CRISPR editing, however, can develop resistant varieties without significantly compromising yield by targeting and modifying specific VQ genes, partially resolving this contradiction [58,59]. Collectively, these findings underscore the pivotal role of VQ proteins in plant stress adaptation and provide a robust molecular framework for their utilization in molecular design breeding.

## Data Availability

The original contributions presented in this study are included in the article. Further inquiries can be directed to the corresponding authors.

## Abbreviations

VQ	Valine-glutamine motif proteins
WRKY	WRKY transcription factors
MAPK	Mitogen-activated protein kinases
DBD	DNA-binding domains
IDR	Intrinsic disordered regions
SIB1	Sigma factor binding protein 1
ROS	Reactive oxygen species
ABA/SA	Abscisic acid/salicylic acid
VQ	Valine-glutamine
P5CS	D-1-pyrroline-5-carboxylate synthetase
WGD	Whole-genome duplication
CAT	Catalase
SOD	Superoxide dismutase
POD	Peroxidase
JA	Jasmonate
JAZ	Jasmonate ZIM-domain
PAMPs	Pathogen-associated molecular patterns
PR	Pathogenesis-related
GLS	Glomerella leaf spot
IDPs	Intrinsically disordered proteins
DPs	Diterpenoid phytoalexins
